# Simple Plug‐In Synthetic Step for the Synthesis of (−)‐Camphor from Renewable Starting Materials

**DOI:** 10.1002/cbic.202100187

**Published:** 2021-06-15

**Authors:** Elia Calderini, Ivana Drienovská, Kamela Myrtollari, Michaela Pressnig, Volker Sieber, Helmut Schwab, Michael Hofer, Robert Kourist

**Affiliations:** ^1^ Institute of Molecular Biotechnology Graz University of Technology Petersgasse 14 8010 Graz Austria; ^2^ Henkel AG & Co. KGaA Adhesive Research/Bioconjugates Henkelstr. 67 40191 Düsseldorf Germany; ^3^ Chemistry of Biogenic Resources Technical University of Munich Schulgasse 16 94315 Straubing Germany; ^4^ Bio, Electro and Chemocatalysis BioCat Fraunhofer Institute for Interfacial Engineering and Biotechnology Schulgasse 11a 94315 Straubing Germany

**Keywords:** asymmetric catalysis, borneol, camphor, esterases, kinetic resolution

## Abstract

Racemic camphor and isoborneol are readily available as industrial side products, whereas (1*R*)‐camphor is available from natural sources. Optically pure (1*S*)‐camphor, however, is much more difficult to obtain. The synthesis of racemic camphor from α‐pinene proceeds via an intermediary racemic isobornyl ester, which is then hydrolyzed and oxidized to give camphor. We reasoned that enantioselective hydrolysis of isobornyl esters would give facile access to optically pure isoborneol and camphor isomers, respectively. While screening of a set of commercial lipases and esterases in the kinetic resolution of racemic monoterpenols did not lead to the identification of any enantioselective enzymes, the cephalosporin Esterase B from *Burkholderia gladioli* (EstB) and Esterase C (EstC) from *Rhodococcus rhodochrous* showed outstanding enantioselectivity (E>100) towards the butyryl esters of isoborneol, borneol and fenchol. The enantioselectivity was higher with increasing chain length of the acyl moiety of the substrate. The kinetic resolution of isobornyl butyrate can be easily integrated into the production of camphor from α‐pinene and thus allows the facile synthesis of optically pure monoterpenols from a renewable side‐product.

## Graphical abstract

**Enzymes are a valuable** tool for upcycling side‐stream from renewable sources. We have found that EstB from *Burkholderia gladioli* could achieve high enantiopurity in upcycling α‐pinene, a side‐product from turpentine production, whereas common lipases and esterases could not. This reaction can be easily integrated with the existing chemical synthetic route for the production of the racemic camphor with minimal adjustments as the route goes through an intermediate isobornyl acetate.

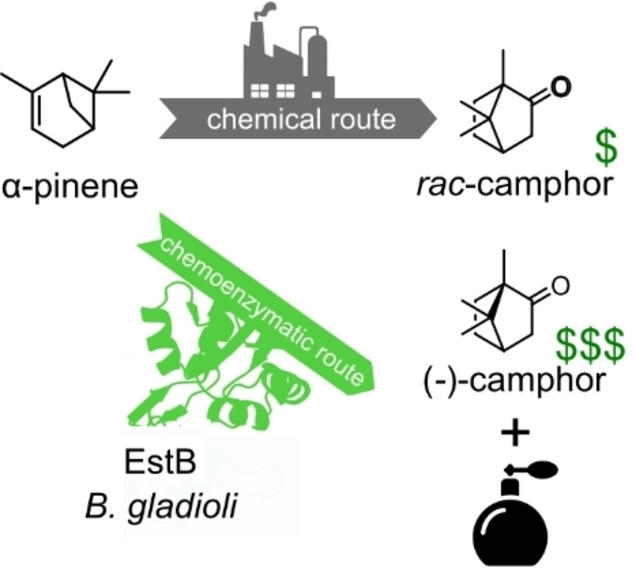

## Introduction

**Scheme 1:**
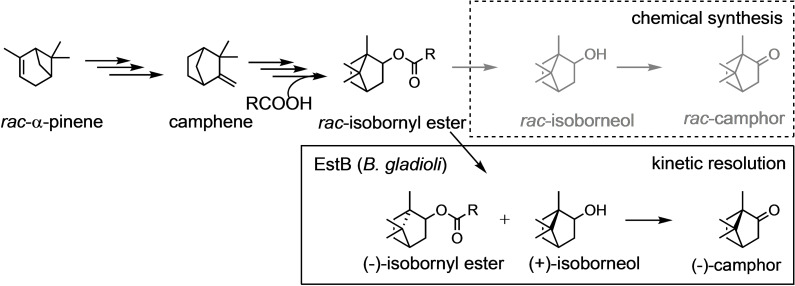
Synthesis route for the production of racemic camphor from α‐pinene.^[11]^ Esterase‐catalyzed kinetic resolution of bornyl esters would substitute non‐selective ester hydrolysis and allow the isolation of both isomers in pure form.

Enzyme catalysis is an efficient and environmentally friendly method for the production and modification of bio‐based chemicals.^[1]^ Furthermore, the demand for enantiopure compounds and synthetic pathways to access single enantiomers is constantly increasing in the chemical and pharmaceutical industry due to stricter requirements by the national regulatory agencies.[^2^, ^3^] In this context, the intrinsic selectivity of enzymes at mild conditions is generally an advantage over organometallic catalysts and other chemo‐catalytic methods for asymmetric synthesis.^[2]^ Several monoterpenes accumulate as side‐products during processes such as the cellulose production from conifers and thus are considered a promising feedstock for the synthesis of chemical products from renewable resources as a substitute for petrol‐based chemicals.^[4]^ In particular, the monoterpenoids isoborneol, borneol, and camphor find application in many products such as food flavoring, cosmetics, and cleaning products.^[5]^ Diverse biological activities such as anti‐inflammatory, vasorelaxant and neuroprotective, among others, make them valuable ingredients for health‐related formulations.^[6]^ The monoterpene camphor and its corresponding alcohols of camphor, borneol, and isoborneol, find application as fragrances and in traditional Chinese medicine. The pure enantiomers of isoborneol and borneol are frequently found in essential oils from many plants, and they have shown a wide array of biological and antimicrobial activities.[^7^, ^8^] Derivatives of these monoterpenoids also find application as chiral ligands in asymmetric synthesis.[^9^, ^10^]

(+)‐camphor (or (1*R*)‐camphor) can be isolated from various natural sources and is widely applied in cosmetics and natural medicine, whereas racemic camphor can be obtained from α‐pinene, a side‐product from turpentine production. Isomerization of α‐pinene yields camphene and afterward, the addition of organic acids to camphene leads to isomerization and the formation of *rac*‐isobornyl esters. Finally, hydrolysis and oxidation yield *rac*‐camphor (Scheme 1).^[11]^ (−)‐Camphor is a constituent of essential oils of plants from the genus Matricaria. The synthesis of pure ingredients of essential oils is a strategy to alter the composition and thus their olfactory properties. The current synthesis of (−)‐camphor proceeds from optically pure alpha‐pinene, which itself must be isolated from plants.

Chemical reduction of *rac*‐camphor produces a mixture of racemic borneol and isoborneol which is called ‘synthetic borneol.^[12]^ Reduction of (+)‐camphor leads to a mixture of (+)‐borneol and (−)‐isoborneol. As the separation of the isomers is costly, they are usually marketed as a mixture under the assignation ‘semisynthetic borneol’.^[13]^ Pure (*1R*)‐borneol can be extracted from the resin and essential oil of woody plants of the families *Dipterocarpaceae*, *Lamiaceae*, *Valerianaceae*, and *Asteraceae*. Borneol and isoborneol can be easily oxidized to camphor chemically or enzymatically.[^11^, ^14^] Pure (−)‐borneol can be isolated from the herbaceous plant *Blumea balsamifera*.^[15]^ While (+)‐camphor and *rac*‐camphor are easily available, (−)‐camphor is more difficult to obtain as it is produced by reduction of (−)‐borneol which can only be extracted from plants.[^15^, ^16^] Its scarce availability and high price make its synthesis an interesting goal.

Previously, we isolated the first enantioselective borneol‐dehydrogenases from Salvia officinalis L.^[17]^ While these enzymes showed high enantioselectivity in the kinetic resolution of borneol and isoborneol, the necessity to recycle the NAD^+^‐cofactor represents a disadvantage. We envisioned that a hydrolase could catalyze the kinetic resolution either with a selective transesterification of isoborneol or with selective hydrolysis of an isobornyl ester. Resolution of norborneol and similar compounds by microbial wild type strains has been reported,^[18]^ but to our knowledge, no isolated enzymes with satisfactory selectivity have been reported for this reaction. Here we report screening and identification of a highly selective esterase for the kinetic resolution of isobornyl butyrate. The esterase‐catalyzed resolution can substitute the non‐selective hydrolytic step in the existing synthetic route from α‐pinene to camphor, thus providing a facile procedure for the manufacture of pure (1*S*,2*S*,4*S*)‐(+)‐isoborneol and (1*R*,2*R*,4*R*)‐(−)‐isobornyl butyrate.

## Results and Discussion

### Kinetic resolution using lipases

Several α/β‐hydrolase‐fold class enzymes are known to accept esters from very bulky alcohols as substrates. This includes hydrolases with a particularly large active site such as lipase A from *Pseudozyma antarctica* (previously *Candida antarctica*), the cephalosporine‐esterase EstB from *Burkholderia gladioli*,^[19]^ and several esterases having a so‐called GGG(A)X‐motif in their active sites can convert tertiary alcohols and their esters.^[20]^ Yet, the selectivity towards sterically hindered substrates is often very low and in most cases hard to predict. At first, 10 commercially available lipases were screened for the enantioselective conversion of borneol or isoborneol by transesterification using vinyl butyrate as acyl donor.^[21]^ This set includes several widely applied lipases and is thus representative of commercially used lipases. For instance, lipases A and B from the yeast *Candida antarctica* are generally cheap and robust catalysts with high activity and selectivity in a large number of classic and dynamic kinetic resolutions.^[22]^ None of the tested lipases showed any significant selectivity toward *rac*‐borneol, resulting in *ee*_P_‐values from below 2 %. Generally, a conversion lower than 15 % was observed even at high enzyme loadings (SI, Figure S2, A and B). Similarly, no satisfactory selectivity was achieved in the kinetic resolution of isoborneol, however, higher conversions were achieved compared to the kinetic resolution of borneol (SI, Figure S2C). The screening of commercial catalysts underlined the difficulty to identify suitable hydrolases for the bulky monoterpenols.

### Kinetic resolution using esterases

In a prescreen of a collection of 15 bacterial esterases in the conversion of sterically demanding esters, five had shown activity towards monoterpenes such as 5‐endo‐norbonen‐2‐yl acetate or tetrahydrofuran‐3‐acetate (data not shown). As 5‐endo‐norbonen‐2‐yl acetate shares the same bicyclic structure as esters from isoborneol and borneol, the five esterases that converted this substrate were tested in the conversion of the α‐acetate or butyrate esters of isoborneol and borneol (Table 2). In a pre‐screen by thin‐layer chromatography, two out of five of the tested esterases, EstB from *Burkholderia gladioli* and EstC from *Rhodococcus rhodochrous*, showed activity in the hydrolysis of bornyl and isobornyl acetate, respectively (data not shown). For EstB, we investigated the wild‐type enzyme and mutant EstB NK70 (originally mutant 214_27). This variant with seven amino acid substitutions was generated by directed evolution and contains the mutation A311V, which is believed to stabilize a relatively unstable loop. EstB NK70 has increased temperature stability (40‐fold higher activity and a 6‐fold longer half‐life time at 45 °C) and higher tolerance against water‐soluble co‐solvents, which we considered advantageous in view of a later implementation of the kinetic resolution.^[23]^ After the identification of active enzymes, we then proceed to determine the preference of the enzymes towards *p*‐nitrophenol esters of different lengths (C2, C4, C5, C6). This revealed a clear preference of EstB and EstB NK70 towards shorter esters whereas EstC seems to prefer longer ones (SI, Figure S1). The low activity of EstB towards *p*‐nitrophenyl hexanoate was a bit surprising given that the enzyme shows excellent activity towards Cephalosporin C, a carboxyl amide with a somewhat larger acyl moiety than hexanoic acid.^[23]^

In the kinetic resolution of racemic esters of isoborneol and borneol, all esterases except the commercial esterase from porcine liver showed better selectivity with butyryl moiety compared to the shorter chain acetate moiety, resulting in up to 7‐fold higher E values towards the butyryl esters. Furthermore, both EstB variants showed high enantioselectivity in the kinetic resolution of isobornyl butyrate, whereas EstC showed high selectivity for bornyl butyrate (Table 1). Interestingly, EstB converted all substrates but only showed selectivity for two, EstC, in contrast, showed no conversion with isobornyl and fenchyl esters as substrate. Conversion of isobornyl hexanoate by EstB, its variant NK70 and EstC yielded no product (data not shown), which was surprising since the corresponding *p*‐nitrophenyl ester was converted, albeit with much lower activity than esters of shorter acids.

**Table 1:** Kinetic resolution of bornyl butyrate and isobornyl butyrate using 1 mM substrate at 30 °C and 600 rpm. Data reported correspond to values obtained at 24‐h intervals. Only esterases with detectable activity are listed.

Substrate	Enzyme	Origin	Conversion^[a]^ (%)	*ee*_S_^[b]^ (%)	*ee*_P_^[a]^ (%)	E‐value^[c]^	Main product enantiomer
*rac*‐**2 a**	Esterase	Porcine liver	49.4 %±4.3 %	0.3 %	0.3 %	1	n.d.
*rac*‐**2 b**	Esterase	Porcine liver	59.4 %±1.2 %	0.2 %	0.1 %	1	n.d.
*rac*‐**1 a**	Esterase	Porcine liver	43.7 %±0.1 %	62 %	80 %	17	(−)‐**1**
*rac*‐**1 b**	Esterase	Porcine liver	42.6 %±1.7 %	16 %	21 %	2	(−)‐**1**
*rac*‐**2 a**	EstB	*B. gladioli*	34.2 %±7.2 %	16 %	31 %	2	(+)‐**2**
*rac*‐**2 b**	EstB	*B. gladioli*	90.4 %±1.9 %	94 %	10 %	3	(+)‐**2**
*rac*‐**1 a**	EstB	*B. gladioli*	16.9 %	19 %	91 %	26	(+)‐**1**
*rac*‐**1 b**	EstB	*B. gladioli*	31.2 %±0.1 %	44 %	98 %	>100	(+)‐**1**
*rac*‐**3 b**	EstB	*B. gladioli*	18 %±0.03 %	20 %	90 %	23	(+)‐**3**
*rac*‐**2 a**	EstB NK70	*B. gladioli*	24.8 %±2.0 %	10 %	31 %	2	(+)‐**2**
*rac*‐**2 b**	EstB NK70	*B. gladioli*	65.5 %±1.2 %	70 %	37 %	4	(+)‐**2**
*rac*‐**1 a**	EstB NK70	*B. gladioli*	23.2 %±0.1 %	27 %	91 %	30	(+)‐**1**
*rac*‐**1 b**	EstB NK70	*B. gladioli*	31.2 %±0%	44 %	98 %	>100	(+)‐**1**
*rac*‐**2 a**	EstC	*R. rhodochrous*	11.2 %±1.5 %	11 %	89 %	19	(−)‐**2**
*rac*‐**2 b**	EstC	*R. rhodochrous*	31.8 %±0.1 %	45 %	97 %	>100	(−)‐**2**

[a] Determined by chiral gas chromatography. [b]  Calculated from the formula: C=ee_s_/(ee_s_+ee_p_), where C corresponds to conversion. [c] Calculated from conversion and ee_P_ according to Chen *et al*.^[24]^

**Scheme 2:**
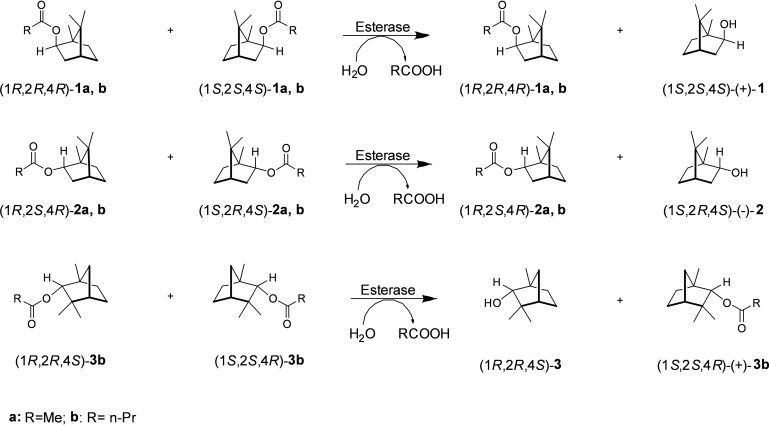
Enantioselective kinetic resolution of racemic acetyl and butyryl esters of isoborneol (**1**), borneol (**2**), and fenchol (**3**) employing esterases.

Surprisingly, EstB from *B. gladioli* showed high activity and excellent enantioselectivity in the kinetic resolution of *rac*‐isobornyl butyrate giving rise to (+)‐isoborneol (or (1*S*,2*S*,4*S*)‐1,7,7‐trimethyl‐bicyclo[2.2.1]heptan‐2‐ol in high optical purity (>98 % *ee*, Scheme 2). Hydrolysis of *rac*‐isobornyl acetate produced (+)‐isoborneol in 89 % *ee* (Scheme 2). The oxidation of (+)‐isoborneol is simple and gives rise to (−)‐camphor (or (1*S*)‐camphor). In contrast, EstC showed high enantioselectivity towards *rac*‐bornyl butyrate and produced (−)‐borneol in 97 % *ee*. From *rac*‐bornyl acetate, (−)‐borneol was produced with 90 % *ee*.

Investigation of a representative set of 10 α/β‐hydrolase‐fold lipases and four α/β‐hydrolase‐fold esterases showed that none of these enzymes had an enantioselectivity level that would suffice for kinetic resolution of isoborneol esters. For such an application, enantioselectivity of E≥20 is the minimum, albeit at this enantioselectivity level the final yield of the optically pure product of interest would be well below 50 %.^[25]^

Esterase B from *B. gladioli* is known to convert esters of bulky alcohols (such as linalyl acetate),^[19]^ EstB differs structurally from the classic esterases and lipases and exhibits homology with esterases of class VIII and with class C β‐lactamases.^[26]^ The natural substrate is presumably Cephalosporin C, a carboxyl amide. Moreover, its catalytic machinery differs considerably from α/β‐fold esterases. This regards the position of the catalytic serine 105 and the nature of the catalytic triad, which does not consist of the typical motif Ser‐His‐Asp/Glu, but of Ser105, Lys78, and/or Trp348.^[26]^ While the enzyme has been shown to accept sterically very demanding tertiary alcohols, the selectivity towards all racemic substrates investigated has been low so far.^[19]^ Surprisingly, this unique hydrolase was the only one from a representative set of lipases and esterases that showed high enantioselectivity (E>100) in the kinetic resolution of isoborneol esters, respectively. It is well‐known that many esterases and lipases show higher enantioselectivity and activity towards esters with longer chains of the same alcohol.^[27]^ This empiric rule is confirmed by EstB and EstC that show both much higher enantioselectivity towards the butyrate ester than towards the acetate esters of isoborneol and borneol, respectively. Interestingly, compared to EstC, EstB shows inverse enantiopreference towards esters from borneol and isoborneol as it preferentially produces (+)‐borneol from racemic bornyl butyrate, whereas it produces (+)‐isoborneol from racemic isobornyl butyrate. The different *endo*‐ and *exo*‐position of the ester group in α‐position of the bicyclic molecules leads to an inversion of the preference of the enzyme. A similar inverse preference towards these bicyclic monoterpenoids was observed in the stereoselective oxidation of borneol and isoborneol by plant dehydrogenases.^[17]^

Implementation of enzyme catalysis can either replace one or more existing steps of a given synthetic route, or a new synthetic route is assembled “*de novo*”. The latter is typically hard to implement as it requires a complete redesign of a certain chemical process, whereas replacing only one step is considerably easier. While the yield limitation of kinetic resolutions is considered a disadvantage, it is still very popular in industrial processes due to the extremely high simplicity.^[28]^ The EstB‐catalyzed kinetic resolution process can be easily integrated into an existing chemical route. In this particular case, the resulting isobornyl and bornyl esters are both used as fragrances, in food preparations and as chiral ligands, or they can be hydrolyzed and oxidized to produce the natural (+)‐camphor and (−)‐camphor isomers. The biocatalytic step simply substitutes the unselective chemical hydrolysis. Unlike oxidoreductases, the esterase does not require the supply of external cofactors or additional steps for their regeneration. This work underlines the simplicity of this approach where the production of the highly valuable (1*S*)‐camphor using an enzyme can outweigh the cost of chromatographic separation of enantiomers. Racemic borneol and bornyl acetate as starting materials for EstC are not readily available but are common compounds in turpentine from different trees and can be isolated during the refinement.[^29^, ^30^]

From an industrial application point of view, the corresponding acetate substrates are more attractive giving that isobornyl acetate is already an intermediate in the chemical synthesis of racemic camphor.^[11]^ We expect that the selectivity of the enzymes towards the acetyl esters can be increased by protein engineering.^[20]^ Nevertheless, butyric acid is an easily available building block that can be obtained both from non‐renewable and renewable resources, and giving its high chemical similarity with acetate we envision that the current process conditions to produce isobornyl acetate will apply to the product of isobornyl butyrate with minor adjustments. The inexpensive isobornyl esters can be then used as substrates for EstB from *B. gladioli* followed by the further oxidation of (+)‐isoborneol to the highly valuable (−)‐camphor. In contrast, EstC from *R. rhodochrous* can catalyze the kinetic resolution of the bornyl esters affording (−)‐borneol which can be oxidized to the valuable (−)‐camphor as well.

Turpentine oils, a waste by‐product from industrial wood processing were identified as possible substituents for fossil‐derived chemicals.[^31^, ^32^] The identification of stereoselective enzymes for the synthesis of high‐value compounds from bio‐based side‐stream can substitute petrol chemicals and the isolation of natural product from plants (which is problematic due to considerable accumulation of waste), respectively, and makes thus a contribution towards the development of clean and economically successful processes.

## Conclusion

We identified two highly selective esterases for the kinetic resolution of isobornyl and bornyl esters. EstB from *B. gladioli* is particularly interesting as it is highly specific for isobornyl butyrate, an intermediate of the chemical synthesis of camphor from α‐pinene. We showed that achieving conversion and high selectivity with these bulky esters is a complex task and none of a representative set of commercially used α/β‐hydrolase‐fold lipases showed mentionable selectivity. EstB is an esterase that exhibits homology with esterases of class VIII and with class C β‐lactamases^[26]^ and possibly its particular and unique structure is required to achieve high selectivity with the substrates used in this study.

We anticipate that the integration of the kinetic resolution of isobornyl esters in the current industrial process can be extremely simple and lead to a more valuable product: (−)‐camphor. This work highlights the high potential of biocatalysis for side‐product valorization while not being invasive in the process design.

## Experimental Section

### Materials

All chemicals were purchased from either Sigma Aldrich Chemie (Steinheim, Germany), Alfa‐Aesar (Thermo Fisher (Kandel) GmbH, Germany), c‐LEcta (Leipzig, Germany) unless otherwise stated.

### Enzymes

#### Enzyme production

Esterases were produced in terrific broth (TB) media at 20 °C overnight according to the standard protocol after adding the appropriate antibiotic and inducer listed in Table 2.[^26^, ^33^]

**Table 2:** List of enzymes used in this work and organism of origin.

Enzyme	Organism	Plasmid/Antibiotic/Inducer
Esterase	Porcine liver	Immobilized (Commercial)
Esterase EstB	*Burkholderia gladioli*	pK214_estB (wt)/kan /IPTG
Esterase EstA	*Burkholderia gladioli*	pJexpress401_estA/kan/ARA
Esterase EstA	*Rhodococcus rhodochrous*	pMS470_estA/amp/IPTG
Esterase EstC	*Rhodococcus rhodochrous*	pMS470d8‐estC/amp/IPTG
Esterase EstA	*Arthrobacter nicotianae*	pMS470_estA/amp/IPTG
Esterase EstB mutant^[a]^	*Burkholderia gladioli*	pK214_estB (NK70)/kan /IPTG

[a] Mutations confirmed by sequencing: S17L G132S E251G A311V E316K.

### Synthesis of monoterpenol esters

**Bornyl, isobornyl, and fenchyl esters**: To 1 g of either borneol or isoborneol (50 mM) and dimethylaminopyridine (DMAP, 6 eq.) in dry dichloromethane (130 mL, respectively), acetyl, butyryl, or hexanoyl chloride (4 eq.) was added dropwise and the solution was stirred overnight. The mixture was washed with 1 M HCl (2×60 mL) and distilled water (2×60 mL). The organic layer was dried over anhydrous Na_2_SO_4_ before the solvent was removed under reduced pressure. Products were purified with column chromatography (cyclohexane:ethyl acetate, 95 : 5 for the acetyl esters and 90 : 10 for the butyryl esters) and isolated yields of 70–85 % were reached.^[34]^ Bornyl acetate,^[35]^ bornyl butyrate,^[36]^ isobornyl butyrate,^[37]^ and isobornyl hexanoate^[37]^ were identified based on the ^1^H‐NMR‐spectra from literature.

**Fenchyl butyrate**: To 100 mg of fenchol and dimethylaminopyridine (DMAP, 6 eq.) in dry dichloromethane (13 mL), butyryl chloride (4 eq.) was added dropwise and the solution was stirred overnight. The mixture was washed with 1 M HCl (2×6 mL) and distilled water (2×6 mL). The organic layer was dried over anhydrous Na_2_SO_4_ before the solvent was removed under reduced pressure. The product was purified with column chromatography (cyclohexane:ethyl acetate, 97 : 3) and an isolated yield of 60 % was reached. Fenchyl butyrate was identified based on the ^1^H‐NMR‐spectra from literature.^[38]^

### Enzymatic kinetic resolution with esterases

All kinetic resolutions were carried out using 1 mM of either bornyl, isobornyl or fenchyl esters in 50 mM potassium phosphate buffer pH 7.5 at 30 °C for 24 h with stirring at 600 rpm. All esterases described in Table 2 were applied as cell‐free extract (9 g/L total protein content). All reactions were carried out in duplicate, samples of 200 μl were periodically taken, extracted with 200 μl of ethyl acetate, dried over sodium sulfate and submitted to GC‐FID analysis.

### *p*‐Nitrophenyl ester assay

In a microtiter plate, cleared cell lysate (10 μL), either directly after cell disruption or after 10‐fold dilution, were added to Tris‐HCl buffer (180 μL 100 mM Tris‐HCl, 150 mM NaCl, pH 7.5). Absorbance over time (λ=410 nm) was measured in the spectrophotometer (BioTek, USA) immediately after the addition of 10 μL of 20 mM DMSO stock of one of the *p*‐nitrophenyl esters: acetate, butyrate, valerate, or hexanoate. The esterase activity in CFE was calculated based on the calibration as the initial reaction velocity (μmol/min). The extinction coefficient calculated for p‐nitrophenolate with the calibration curve at the stated pH and buffer was 17700 M/cm.

## Conflict of interest

The authors declare no conflict of interest.

## Supporting information

As a service to our authors and readers, this journal provides supporting information supplied by the authors. Such materials are peer reviewed and may be re‐organized for online delivery, but are not copy‐edited or typeset. Technical support issues arising from supporting information (other than missing files) should be addressed to the authors.

Supporting Information
