# Supplementary material for: Simple Plug‐In Synthetic Step for the Synthesis of (−)‐Camphor from Renewable Starting Materials
Source: Chembiochem. 2021 Jun 15;22(20):2951–6. doi: 10.1002/cbic.202100187 (PMC8596451; doi:10.1002/cbic.202100187)
Supplement: Supplementary file 1 — Supporting Information [file CBIC-22-2951-s001.pdf]

ChemBioChem

Supporting Information

Simple Plug-In Synthetic Step for the Synthesis of (−)-Camphor from Renewable Starting Materials

Elia Calderini, Ivana Drienovská, Kamela Myrtollari, Michaela Pressnig, Volker Sieber, Helmut Schwab, Michael Hofer,* and Robert Kourist*

Supporting methods

Conversion of optically pure borneol enantiomers using lipases

All biotransformations were carried out in 1 mL anhydrous *tert*-butylmethylether (tBME) using 5 mM of either (+)- or (-)-borneol, 3 equivalent of vinyl butyrate (15 mM), and 4Å molecular sieves. 10 mg/mL or 50 mg/mL of each immobilized commercial lipase listed in Table S1 was added and the reactions were carried out at 30 °C. 300 µl samples were periodically taken, dried over anhydrous sodium sulfate, and then 200 µL of the supernatant were submitted to GC-FID for further analysis.

All lipases used in this study are commercially available immobilized lipases ordered from Sigma-Aldrich Gmbh or c-LEcta jointly developed and co-marketed with Purolite. Details on the organisms they originated from, manufacturer, CAS, and product number are presented in the following table.

Table S1. List of commercially available lipases screened in this study.

Enzyme	Organism	Plasmid / Antibiotic / Inducer	CAS	Product Number	Company
1 - Lipase	Hog pancreas	Immobilised (Commercial)	9001-62-1	62300	Sigma-Aldrich
2 - Lipase (CAL-A)	*Candida antarctica*	Immobilised (Commercial)	9001-62-1	02569	Sigma-Aldrich
3 - Lipase (NZ435 CAL-B)	*Aspergilllus niger*	Immobilised (Commercial)	9001-62-1	L4777	Sigma-Aldrich
4 - Amano lipase	*Burkholderia cepacia*	Immobilised (Commercial)	-	534641	Sigma-Aldrich
5 - Lipase	*Penicillium camemberti*	Immobilised (Commercial)	9001-62-1	96888	Sigma-Aldrich
6 - Lipase	*Aspergilllus niger*	Immobilised (Commercial)	9001-62-1	62301	Sigma-Aldrich
7 - IMMO + (CAL-B)	*Candida antarctica*	Immobilised (Commercial)	-	20606-4	c-LEcta-Purolite
8 - IMMO T2	*Candida antarctica*	Immobilised (Commercial)	-	54326	Sigma-Aldrich
9 - Lipase	*Burkholderia cepacia*	Immobilised (Commercial)	9001-62-1	62309	Sigma-Aldrich
10 - Lipase	Porcine pancreas	Immobilised (Commercial)	9001-62-1	62300	Sigma-Aldrich

Supporting figures

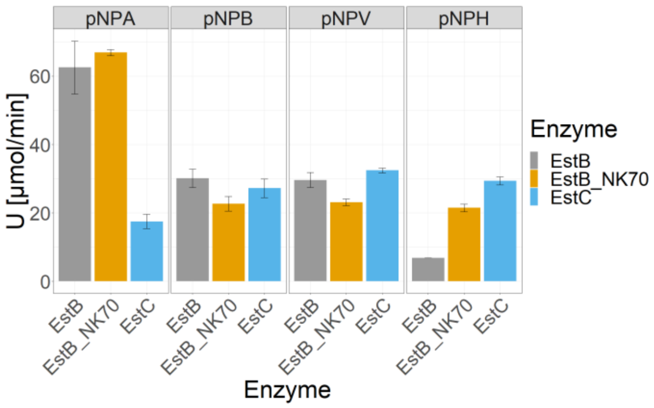

Figure S1. Activity measurements for the EstB and EstC using different p-nitrophenyl esters: acetate (pNPA), butyrate (pNPB), valerate (pNPV) and hexanoate (pNPH). All measurements were carried out in triplicate. A unit is defined as 1 µmol of *p*-nitrophenol produced per minute.

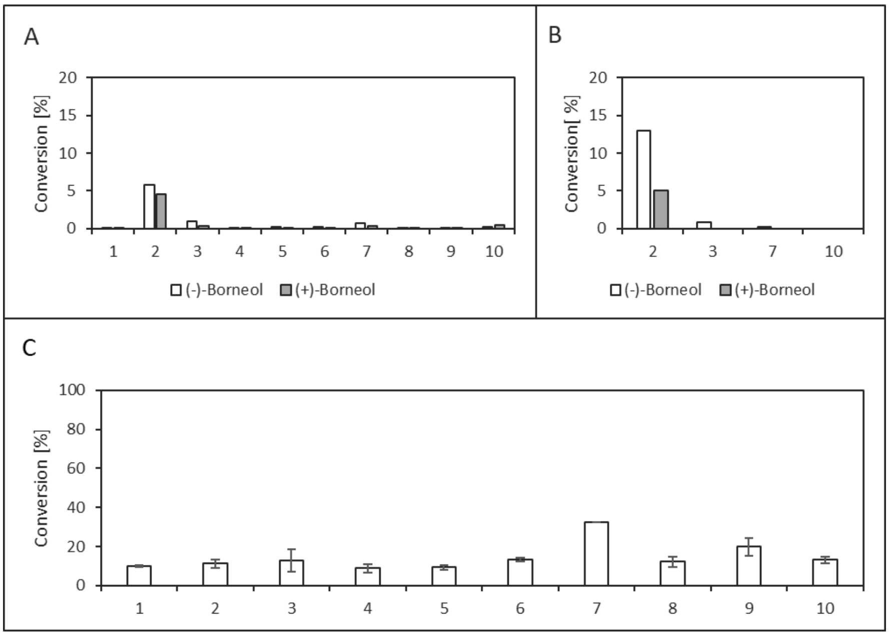

Figure S2. Results obtained from the screening of 10 commercially available lipases (Table S1) using 10 mg/mL of immobilized enzyme and 5 mM of each borneol enantiomer (A). For lipases showing very low activity, higher enzyme loadings were tested (50 mg/mL) (B). For the kinetic resolution of *rac*-isoborneol employing the 10 lipases considered in this study, the ee_P% varied between 0 and ±2% (C).

GC-FID Analysis

Quantitative GC-FID Analysis. GC-FID (GC-2030 Plus, Shimadzu, Japan) equipped with a chiral column was used to analyze samples taken from biotransformations. Sample preparation was carried out by organic phase extraction using ethyl-acetate. Before implemented to GC-FID, samples were dried over anhydrous Na_2SO_4, centrifuged at 12.000 rpm for 10 min, and then the supernatant was transferred to 1.5 mL vials equipped with 200 µL inserts. The methods used for the analyses are described in detail in Table S2.

Table S2. Methods used for monitoring the biotransformations. Method II was used for the efficient separation of the hydrolysis products of fenchyl butyrate. Efficient separation of the hydrolysis products of the rest of the substrates was achieved using Method I.

**Parameters**	**Method I**	**Method II**
**Column**	Hydrodex-β-6TBDM chiral column (25 m, 0.25 mm I.D., 0.25 µM df)	Hydrodex-ß-TBDAc chiral column (50 meter, 0.25 mm ID, 0.25 µm df)
**Flow Control Mode**	Linear velocity (30.1 cm s^{-1}), Carrier gas: N_2	Linear velocity (30.1 cm s^{-1}), Carrier gas: N_2
**Total flow**	115.6 mL min^{-1}	154.3 mL min^{-1}
**Column flow**	1.11 mL min^{-1}	1.5 mL min^{-1}
**Injection Mode**	split	split
**Pressure**	76.4 kPa	158.6 kPa
**Split ratio**	100.0	100.0
**Temperature program**	60 °C for 8 min, 2 °C min^{-1} to 150 °C, 45 °C min^{-1} to 200 °C<br>Total program time: 56.11 min	60 °C for 8 min, 2 °C min^{-1} to 190 °C, 45 °C min^{-1} to 200 °C<br>Total program time: 75.22 min

GC-FID chromatograms

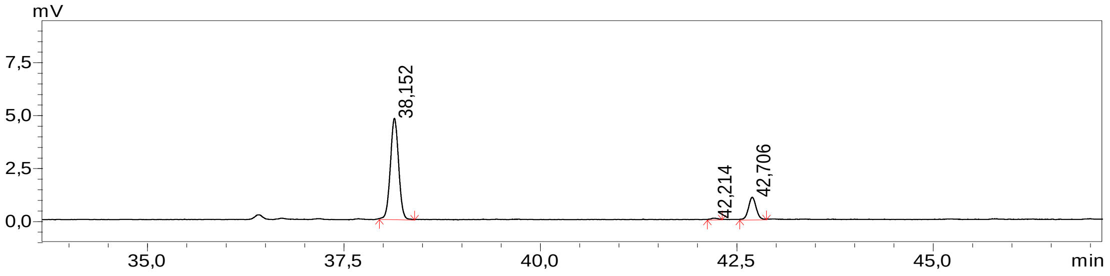

Figure S3. GC-FID chromatogram obtained from the hydrolysis of **1a** (t_R= 38.2) in the presence of EstB after 24h. Retention times of hydrolysis products: *S*-enantiomer 42.2 min, *R*-enantiomer 42.7 min.

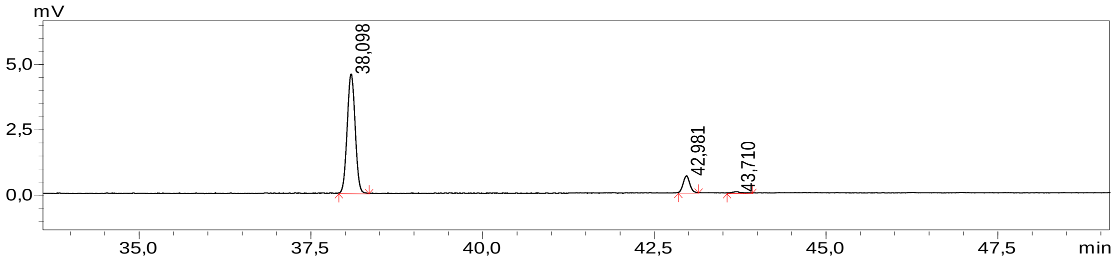

Figure S4. GC-FID chromatogram obtained from the hydrolysis of **2a** (t_R= 38.1) in the presence of EstC after 24h. Retention times of hydrolysis products: *S*-enantiomer 42.9 min, *R*-enantiomer 43.7 min.

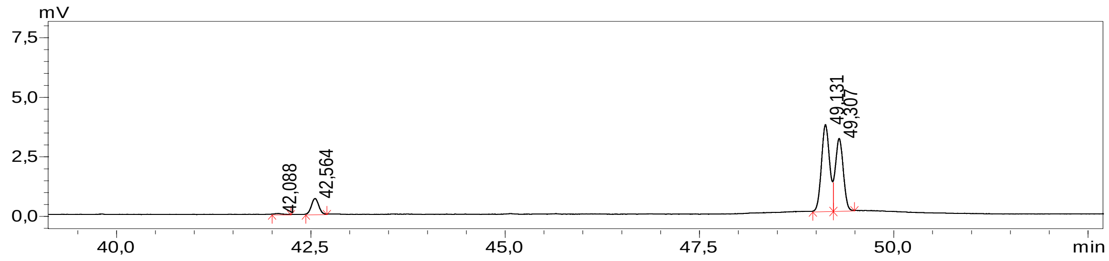

Figure S5. GC-FID chromatogram obtained from the hydrolysis of **1b** (*R*-enantiomer: t_R= 49.3, *S*-enantiomer: t_R=49.1) in the presence of EstB NK70 after 24h. Hydrolysis products obtained are efficiently separated (*S*-enantiomer: 42.1 min, *R*-enantiomer: 42.6 min). Interestingly, **1b** seems to be the only substrate where enantiomers can separate, but still, the separation is not efficient enough to calculate eeS.

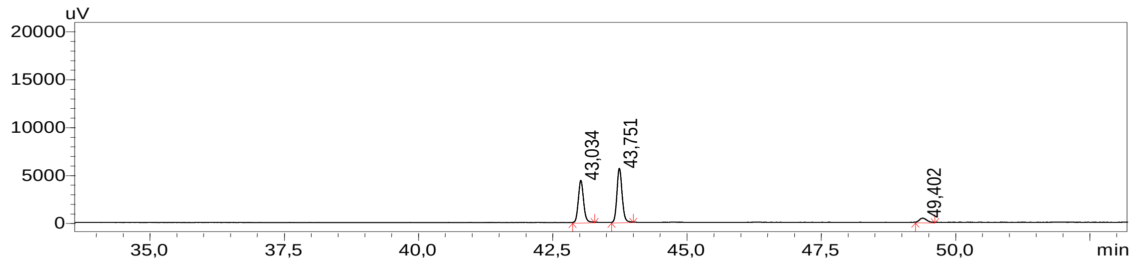

Figure S6. GC-FID chromatogram obtained from the hydrolysis of **2b** (t_R= 49.4) in the presence of EstB after 24h. Retention times of hydrolysis products: *S*-enantiomer 43.0 min, *R*-enantiomer 43.8 min.

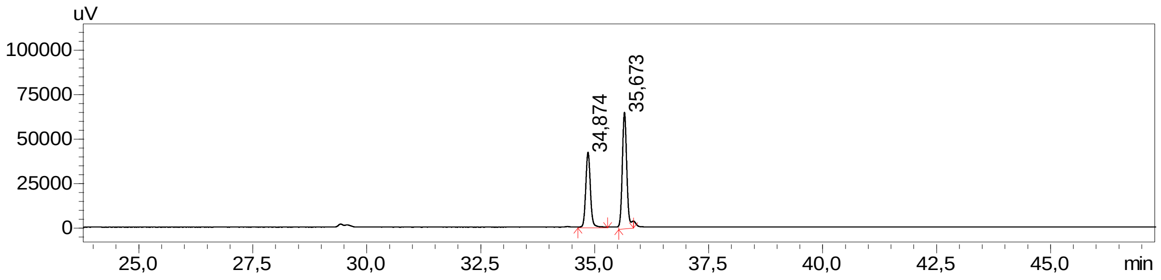

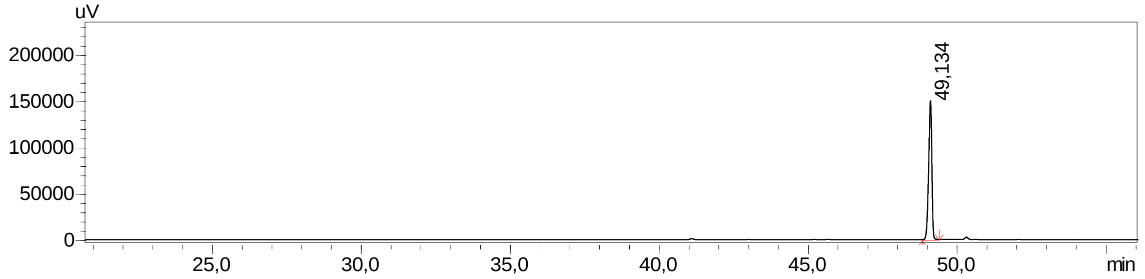

Figure S7. Top: GC-FID chromatogram of commercially available alcohol (*S*-enantiomer: 34.9 min, *R*-enantiomer: 35.7 min). Bottom: GC-FID chromatogram of the synthesized **3b.** *R*- and *S*- enantiomers didn't separate in any of the tested methods. This method was chosen for further experiments due to the efficient separation of the hydrolysis products.
